# Reply to Letter to the Editor by Yin et al.

**DOI:** 10.1111/irv.12895

**Published:** 2021-08-10

**Authors:** Brenda L. Coleman, Ruth Sanderson, Mendel D. M. Haag, Ian McGovern

**Affiliations:** ^1^ Sinai Health System Toronto ON Canada; ^2^ Dalla Lana School of Public Health University of Toronto Toronto ON Canada; ^3^ Western University London ON Canada; ^4^ Seqirus NL BV Amsterdam The Netherlands; ^5^ Seqirus Inc. Cambridge Massachusetts USA

**Keywords:** influenza, systematic review, vaccines

## CONFLICT OF INTEREST

BLC and RS were employed by Sinai Health which received funding from Seqirus for this review. MDMH and IM are employed by Seqirus.

## AUTHOR CONTRIBUTIONS

**Brenda Coleman:** Data curation; formal analysis; investigation; methodology; project administration; resources; supervision; validation. **Ruth Sanderson:** Data curation; investigation. **Mendel Haag:** Conceptualization; methodology; project administration. **Ian McGovern:** Conceptualization; data curation; methodology; project administration; visualization.

### PEER REVIEW

The peer review history for this article is available at https://publons.com/publon/10.1111/irv.12895.

Dear Professor Cowling,

We would like to thank you for the opportunity to address the suggestions raised in the letter by Yin et al. in response to our study “Effectiveness of the MF59‐adjuvanted trivalent or quadrivalent seasonal influenza vaccine among adults 65 years of age or older, a systematic review and meta‐analysis” that found that among adults ≥65 years, aTIV demonstrated significant absolute vaccine effectiveness (VE), improved relative VE compared to non‐adjuvanted standard‐dose TIV/QIV and comparable relative VE to high‐dose TIV.^1^, ^2^ We would also like to thank Yin et al. and his colleagues at Sanofi Pasteur for their interest in our paper and for their appraisal.

The decision to focus on real‐world evidence was based on an interest in synthesizing data on the performance of aTIV/aQIV under real‐world conditions in the general population. Randomized controlled trials (RCTs) are generally considered the “gold standard” of evidence due to their potential to produce relatively unbiased effect estimates. However, clinical trial inclusion/exclusion criteria may result in a study population that is not representative of the general population, potentially decreasing the generalizability of the results to routine care in the general population. Seasonal influenza vaccines are reformulated almost every year due to the constantly changing antigenic properties of circulating influenza viruses. This frequent change necessitates annual evaluation of influenza vaccine performance, which is accomplished primarily though RWE.

One study in our review (Gasparini et al.^3^) was identified as being at serious risk of bias (RoB). A recent review^4^ by the European Centre for Disease Prevention and Control (ECDC) characterized two additional studies as being at serious RoB that were characterized as moderate RoB in our review (Spadea et al.^5^ and Mannino et al.^6^). While the ROBINS‐I tool (and other RoB assessment tools) provides a structured framework for evaluating RoB, a degree of subjectivity remains and variation in the assessment outcomes may be expected between different reviewers/reviews. The original reported pooled estimate for aTIV absolute VE for the prevention of influenza and pneumonia hospitalizations was 51.3% (95% CI: 39.1, 61.1). In a post hoc analysis conducted for this letter, the pooled estimate did not change considerably when the Gasparini 2013 study was removed (50.6% [38.2, 60.6]) or when both the Gasparini et al. and Spadea et al. studies were removed (54.8% [29.1, 71.1]). Similarly, the original pooled estimate for the relative VE of aTIV versus TIV for the influenza‐related medical encounters (13.9% [4.2, 23.5]) did not change considerably when Mannino et al. was removed in a post hoc analysis (13.0% [2.9, 23.0]).

Non‐peer‐reviewed data (commonly referred to as “grey literature”) are frequently included in systematic reviews because it helps to ensure a comprehensive assessment of all available literature and the exclusion of that data can lead to publication bias.^7^ Our study reviewed a wide range of grey literature sources in an effort to identify all available relevant data. The grey literature study noted by Yin et al. (Van Buynder et al.^8^) was a second season extension of a study that was previously published in a peer‐reviewed journal (and included in our review).^9^ A second effect estimate from a grey literature source (Public Health England [PHE] government report)^10^ was also included in the meta‐analysis of absolute VE of aTIV for prevention of lab‐confirmed outpatient influenza visits. The original pooled estimate of the absolute VE of aTIV for prevention of lab‐confirmed outpatient influenza visits was 40.7% (21.9, 54.9) and increased in a post hoc analysis when the Van Buynder et al. study was removed 44.6% (6.7, 67) and when both the Van Buynder et al. and PHE estimates were removed 59.8% (25.8, 78.3), indicating that our reported estimate was more conservative than if grey literature sources had been excluded.

There were additional studies evaluated in our review that were not captured in the reviews conducted by Canada's NACI, the ECDC, or STIKO, predominantly due to the later search cutoff date for our review.^4^, ^11^, ^12^ All four reviews (NACI, ECDC, STIKO and our review) had differences in their specific research question/aim, study selection, and evidence synthesis approaches. The conclusions of the NACI, ECDC and STIKO reviews were based on the results of GRADE assessments.^13^ The GRADE method is a general approach (i.e., not influenza specific) of synthesizing and “grading” the quality of evidence for a certain research question (or questions). The GRADE approach puts a heavy emphasis on RCTs in “grading” the body of evidence with RCTs starting as “high quality” and observational evidence starting as “low quality.” Decision makers may consider a wide variety of factors when evaluating a health technology (e.g., efficacy, effectiveness, safety, cost, and supply), and it is up to those decision makers to decide what weight to give to any one factor. Additionally, the weight any one decision maker may give to RWE when evaluating a health technology does not determine the validity of the available body of RWE evidence or any review/meta‐analysis of that body of evidence. Similar evaluations by the Australia's ATAGI and UK's JCVI led to preferential recommendations for aQIV or high‐dose QIV over standard dose nonadjuvanted egg‐based vaccines for adults aged 65 years or older.^14^, ^15^

The results of our study represented the most up to date body of RWE evidence related to aTIV/aQIV effectiveness (at the time of the review) and give insights into how the aTIV performs in a real‐world setting and population. The potential limitations inherent to observational evidence were acknowledged and discussed in the manuscript. Accounting for the various scenarios suggested by the responding authors did not impact the study's overall conclusion that among adults ≥65 years, aTIV demonstrated significant absolute vaccine effectiveness (VE), improved relative VE compared to nonadjuvanted standard‐dose TIV/QIV, and comparable relative VE to high‐dose TIV.

## Data Availability

This is a letter to the editor. Access to the original data is available as per the original article.
